# Municipal Milk Supply

**Published:** 1904-02-06

**Authors:** 


The Hospital.

A Journal of

The Medical Sciences and Hospital Administration.

Vol. XXXV.—No. 906. FEBRUARY 6, 1904.

### Municipal Milk Supply.

We have often had to call attention to the very valuable papers on various subjects connected with the preservation of public health, which from time to time appear in the Journal of the Sanitary Institute of Great Britain, usually as records of the work of some preceding congress. The concluding part of the volume of the Journal for last year has just been published; and, among a large quantity of interesting material of other kinds, it contains an account of the endeavours which have been made by various municipalities to arrest an excessive infantile mortality by a supply of humanised milk, under safe conditions, at prices within the reach of the wage-earning poor, or by the intervention of the relieving officer when this was required. The principal paper dealing with the subject was read by Dr. McCleary, the medical officer of health for Battersea, and it was followed by an instructive discussion which is also fully reported. Dr. McCleary told his audience that the system was originated by Dr. Leon Dufour, of Fécamp, who opened his institution, the Goutte de Lait, in 1894. Similar Gouttes de Lait have since been opened in many other French towns, some by municipalities, others by philanthropic societies; and the first in this country was established by the Corporation of St. Helen's, in 1899, on the advice of Dr. F. Drew Harris, their Medical Officer of Health. In the following year municipal milk depôts were opened at Liverpool, Ashton-under-Lyne, and Dukinfield, and since then Battersea, Leith, and Bradford, have taken the same step.

The conduct of these several establishments is managed by slightly different methods. At Battersea, the milk is supplied by a local dairyman and arrives in the early morning. Its freedom from chemical preservatives is guaranteed by the vendor, and it must contain not less than 3·25 per cent. of butter fat. It is then prepared in one of three methods, according to the ages of the infants for whom it is designed. The first modification contains two parts of water to one of milk, with an addition of seven ounces of cream and seven ounces of sugar of milk to each gallon of the mixture. This is given to infants under three months old. The second modification, for infants between three and six months old, consists of equal parts of milk and of water, with five ounces of cream and of sugar of milk to the gallon. The third consists of two parts milk to one of water, with three ounces each of cream and of sugar of milk to the gallon, and is given to infants over six months old.

The several mixtures having been prepared each morning in the requisite quantities, they are next bottled in bottles containing from $1\frac{1}{2}$ oz. to 7 oz. in eight gradations of size. The bottles are closely stoppered, and are exposed to steam heat, being kept at a temperature of 212° for from five to ten minutes. They are then allowed to cool, and are ready to be supplied to customers. The mother is instructed, when feeding time arrives, to place an unopened bottle in hot water till the milk has reached body temperature. It is then to be opened, a small teat placed over its mouth, and the child takes its food from the original bottle direct, the use of any intermediate one being avoided. A child under two weeks of age is supplied with nine bottles daily, each holding an ounce and a-half of the appropriate mixture. After the fortnight, the bottles are increased in size to hold two and a-half ounces; and after two months the daily number is diminished to eight, and the size increased to three ounces. There are several other gradations, and ultimately a child between eight and twelve months receives six bottles a day, each holding seven ounces of the third modification.

It is extremely difficult to arrive at any certainty with regard to the results of the system thus briefly described. Dr. McCleary has done his best in this direction; but he has to deal with a very shifting population, and also with the uncertainties which arise from mothers bringing sick children for the municipal milk, almost as they would bring them to a hospital, and falling back upon earlier methods of feeding as soon as their anxieties are relieved. It is also to be remembered that the earliest infantile mortality, that occurring within the first two weeks of life, is usually less dependent upon external conditions than upon maternal weakness or disease, and that these young infants die because many of them are not fit to live. It may perhaps be assumed, as an additional factor in the situation, that the mothers who obtain the municipal milk are likely to be among the better examples of their class, and hence to take more care of their children than would be done by others; but, when all due allowances are made, the experience of Battersea certainly seems to show that the milk supply is both popular and beneficial. Dr. McCleary's paper was followed by a discussion in which the representatives of several important towns took part, and in which it was said on the part of Norwich by Alderman Stevens, the vice-Chairman of the local Health Committee, that his committee had seriously considered the question of a municipal milk supply, but that they had been told, by the medical gentlemen of the city, assembled in conference, that the proposed course was undesirable. They were told that sterilised milk, if continuously given, instead of being a benefit to the recipients, might be injurious, and might lead to various complaints such as rickets and other things. A majority of the medical gentlemen present at the conference decided against sterilised milk, and the Committee abandoned the project accordingly, and had since been seeking for other means by which the general milk supply of the city might be improved. No medical representative of Norwich is recorded to have spoken on the occasion, and no medical speaker made any attempt to defend the position which the Norwich practitioners were alleged to have taken up, and which, we think, may possibly have been in some way mistaken or misrepresented. Sterilised milk is a poor substitute for that of a healthy mother ; but we believe it to have been the means of saving many lives, and that its use, within certain limits, should be definitely encouraged by sanitary authorities.

